# Nordic environmental resilience: balancing air quality and energy efficiency by applying artificial neural network

**DOI:** 10.3389/fpubh.2024.1429058

**Published:** 2024-11-05

**Authors:** Abul Ala Noman, Faheem Ur Rehman, Irfanullah Khan, Mehran Ullah

**Affiliations:** ^1^Faculty of Management and Economics, Ruhr University Bochum, Bochum, Germany; ^2^MEU Research Unit, Middle East University, Amman, Jordan; ^3^Interdisciplinary Research Centre for Digital Economy and Finance, Business School, King Fahd University of Petroleum and Minerals, Dhahran, Saudi Arabia; ^4^Interdisciplinary Research Centre for Smart Mobility and Logistics, King Fahd University of Petroleum and Minerals, Dhahran, Saudi Arabia; ^5^School of Business and Creative Industries, University of the West of Scotland, Paisley, United Kingdom

**Keywords:** environmental management, air quality, energy efficiency, artificial neural network, Nordic countries

## Abstract

Maintaining public health and environmental safety in the Nordic nations calls for a strict plan to define exact benchmarks on air quality and energy efficiency. This study investigates the complicated interaction of decentralized energy production (DEP) with energy efficiency, and air quality index in the Nordic nations from 1990 to 2022 using System GMM and Artificial Neural Network (ANN) approach. Our research explored positive role of decentralized energy production and technological advancement to propel notable increases in energy efficiency, hence lowering pollution expressed as PM2.5 level. Our research indicates, however, that although international trade, GDP and urbanization assist to enhance energy efficiency, they also contribute to pollution by raising PM2.5 Level by higher energy usage. Furthermore damaging to environmental quality is the persistent link shown by economic disparity and the energy price index with increased degrees of pollution and less energy efficiency. Policy frameworks must devised sustainable development policy (decentralized energy production) to significantly improve energy efficiency and lower the amount of pollution. This calls for proper urban planning and a close observation of the possible drawbacks of growing GDP, trade, economic disparity, and energy expenses.

## 1 Introduction

The concerns of air pollution, energy security, and environmental sustainability require urgent and thorough policy responses (1, 2). Within this particular setting, the shift toward decentralized energy production offers a hopeful pathway for effectively tackling these complex difficulties. Decentralized energy systems, which consist of a wide range of renewable sources like wind, solar, and hydropower, have significant potential to not only increase energy efficiency but also enhance air quality (3). The objective of this study is to elucidate the intricate correlation between decentralized energy production and its impact on both energy efficiency and air quality in the Nordic countries, namely Norway, Sweden, Denmark, and Finland.

The deliberate choice to carry out this empirical research in the Nordic nations is purposeful for multiple reasons. Initially, these countries have established significant goals for decreasing greenhouse gas emissions and shifting toward sustainable energy systems (4, 5). Using decentralized energy sources like wind, solar, and hydropower is in line with these objectives and offers the possibility of reducing carbon emissions (5). Norway exemplifies exemplary leadership in the field of renewable energy. Due to its enormous hydropower resources, the country is able to produce a substantial amount of its electricity from renewable sources. This not only decreases carbon emissions but also strengthens energy security and independence (6, 7). Sweden and Denmark have both established varied energy portfolios that encompass renewable sources, fossil fuels, and nuclear energy. The presence of diverse energy sources provides a compelling opportunity to examine the effects of decentralized energy generation on both energy efficiency and air quality (8). The region’s focus on energy efficiency is remarkable in both residential and industrial domains. Sweden has implemented comprehensive energy-efficiency initiatives aimed at enhancing energy efficiency in buildings and enterprises. The European Parliament and the Council have just released Directive (EU) 2024/1275, which aims to enhance the energy efficiency in the European Union in order to attain climate neutrality by 2050 (9, 10).

Furthermore, air quality in the Nordic countries is often acceptable. However, there are specific periods when PM2.5 levels may rise, particularly in urban regions. Recently, there has been more focus on addressing this issue, and Sweden has been particularly noteworthy for its endeavors to decrease air pollution in urban areas (11–13). The current body of research on decentralized energy systems, energy efficiency, and air quality highlights the potential benefits and obstacles involved in shifting toward sustainable, decentralized energy sources. Multiple studies emphasize the capacity of decentralized renewable energy to improve energy efficiency and decrease greenhouse gas emissions (14, 15). Nevertheless, it is imperative to carefully examine the practical consequences of these assertions. An examination of this literature highlights that the success of decentralized energy solutions in enhancing energy efficiency is contingent upon several factors, such as the regulatory framework, technological advancement, energy costs, and trade competitiveness (16–18). Furthermore, although the idea of transitioning to decentralized renewable energy production is often presented as a solution for lowering air pollution, the existing body of research emphasizes the many complexities involved. Several studies have indicated that the production, installation, and preservation of renewable energy infrastructure, such as wind turbines and solar panels, can cause certain environmental effects in the surrounding areas (19, 20). These crucial insights emphasize the necessity for a detailed analysis of the environmental compromises linked to decentralized energy systems. When evaluating renewable technology, policymakers and academics should take into account the comprehensive environmental impact, recognizing that sustainability encompasses more than just carbon emission reduction.

Although there has been extensive research conducted worldwide on the effects of decentralized energy production (DEP) on energy efficiency and environmental quality, there is a lack of empirical studies specifically examining the trade-off between DEP, energy efficiency (EE), and air quality (PM2.5 Level) in a distinct context. Moreover, the Nordic countries have not received adequate attention in recent literature. This empirical study seeks to address this gap by providing a detailed examination of the impact of decentralized energy production on energy efficiency and air quality in the Nordic region. This research is relevant and important, particularly considering the ongoing progress in renewable energy technology and changing environmental issues (4, 21). Nevertheless, it is crucial to recognize the extent and constraints of this study. The energy-environment nexus is a complicated system that encompasses multiple factors and relationships. While this study attempts to address certain important areas, it may not fully encompass all the dynamics involved. Moreover, the empirical results may depend on the particular time period and methodological decisions. The relevance of this research is emphasized by the growing international attention toward renewable energy, sustainability, and efforts to mitigate climate change. The Nordic countries, through their successful implementation of renewable energy integration and energy efficiency measures, have the ability to provide vital insights to the global community.

This paper aims to investigate the impact of decentralized energy production (DEP) on energy efficiency (EE) and PM2.5 pollution levels. The study also incorporate the impact of other economic factors such as Gross Domestic Product (GDPit), urbanization (URBit), technology (TECHit), trade volume (TRADEit), income inequality (INEQit), and the energy price index. The study uses a panel of Nordic countries from 1990 to 2022 and System GMM technique to account for heterogeneity, non-stationarity, and potential endogeneity among the variables and sample units. The study’s findings indicated that decentralized energy production (DEP), urbanization (URB), and technological innovation (TECH) have a substantial positive impact on energy efficiency (EE) and lead to a significant reduction in PM2.5 levels. The findings reveal that gross domestic product (GDP) and trade exert a substantial positive influence on energy efficiency, but they also contribute to the degradation of air quality by increasing PM2.5 pollution as a result of excessive energy consumption. Both income inequality and the energy price index have a detrimental and noteworthy effect on energy efficiency, while also leading to a rise in PM2.5 pollution. In addition to the findings of the System GMM technique, an Artificial Neural Network (ANN) was used to examine the effects of decentralized energy production (DEP) on energy efficiency (EE) and PM2.5 pollution levels in a panel of Nordic countries. Artificial Neural Networks (ANN) are a specific category of machine learning models that draw inspiration from the intricate structure and operation of the human brain. Artificial Neural Networks (ANN) are frequently employed in computer vision for tasks such as Natural Language Processing, picture recognition, speech recognition, and pattern identification (22, 23). ANN has become a commonly utilized machine learning algorithm due to its ability to excel in learning complex and nonlinear mappings.

Overall, the vast body of research on decentralized energy systems, energy efficiency, and air quality provides significant knowledge regarding the possibilities and obstacles associated with shifting toward sustainable energy sources. Nevertheless, a thorough analysis of existing body of literature highlights the necessity of adopting a comprehensive and situation-specific strategy in order to fully exploit the benefits of decentralized energy solutions. To advance the decentralized energy transition, it is crucial to tackle regulatory obstacles, fully comprehend the environmental effects of renewable energy sources, and carefully analyze the complexities of policy implementation.

## 2 Review of literature: the theoretical framework

The current body of literature extensively examines the correlation between decentralized energy production, energy efficiency, and environmental quality. This part will examine the accomplishments of the Nordic nations in the field of decentralized energy systems, emphasize the unique elements of our study, and explore the existing knowledge regarding the correlation between these factors.

The implementation of decentralized energy systems has been shown to have beneficial effects on energy efficiency through the reduction of energy losses and the promotion of renewable energy utilization. Nevertheless, the influence on environmental quality can vary depending on the individual circumstances (24, 25). Although decentralized systems are typically linked to decreased emissions, there may still be localized environmental effects. The implications mentioned could arise from the production and installation of renewable energy infrastructure, as well as the energy-intensive processes involved. Hence, it is crucial to adopt a sophisticated perspective in comprehending the wider consequences of decentralized energy systems on air quality (26, 27).

The Nordic countries, namely Norway, Sweden, Denmark, and Finland, have gained international recognition over the past twenty years for their aggressive initiatives in developing sustainable energy systems, prioritizing environmental quality, and emphasizing decentralized energy generation. The aforementioned countries have demonstrated exemplary utilization of decentralized energy sources, such as solar, wind, and hydropower, to enhance energy efficiency and mitigate greenhouse gas emissions (28, 29).

Previous research has shown that these nations, which are often known for their natural resources, have embraced renewable energy technologies to diversify their energy portfolios. Norway’s extensive use of hydropower and Denmark’s leadership in wind energy are well-documented cases. The literature highlights how these countries have used sources to become prominent in the global transition to renewable energy (16). Sweden, Denmark, and Norway consistently rank high in the Energy Efficiency Index (4). Research has shown that these countries have achieved energy efficiency by adopting sustainable building standards, promoting public transportation, and implementing strict emission control measures. This dedication to energy efficiency is rooted in their shared commitment to sustainable development and adherence to the United Nations’ Sustainable Development Goals.

The manufacturing and installation of renewable energy infrastructure, as well as the energy-intensive processes involved, can have significant environmental implications. As such, it is crucial to take a nuanced approach when studying the broader impacts of decentralized energy systems on air quality, as previous research has shown (30, 31).

Moreover, policy and governance exert a substantial influence on the results of decentralized energy systems. Existing research (16) have observed that the implementation of supportive policies and incentives can effectively promote the adoption of renewable energy technology. Nevertheless, the lack of regulatory flexibility and reluctance to adopt new methods can impede the process of decentralizing energy production. Decentralized energy systems (DES) are now recognized as a crucial approach for tackling the issues of energy security, climate change, and sustainable development. Nevertheless, there is a scarcity of research on DES in the Nordic environment. Across order to fill this void, our research aims to conduct a detailed examination of the distinct dynamics of Distributed Energy Systems (DES) and their consequences for energy efficiency and environmental sustainability across the Nordic nations.

The foundation of our work is rooted in the current body of research that emphasizes the significance of decentralized energy generation in achieving sustainable development (32, 33). Our work adds to the existing body of research by specifically examining the Nordic region, where the implementation of renewable energy and energy efficiency are fundamental aspects of sustainability efforts.

**Figure 1:**
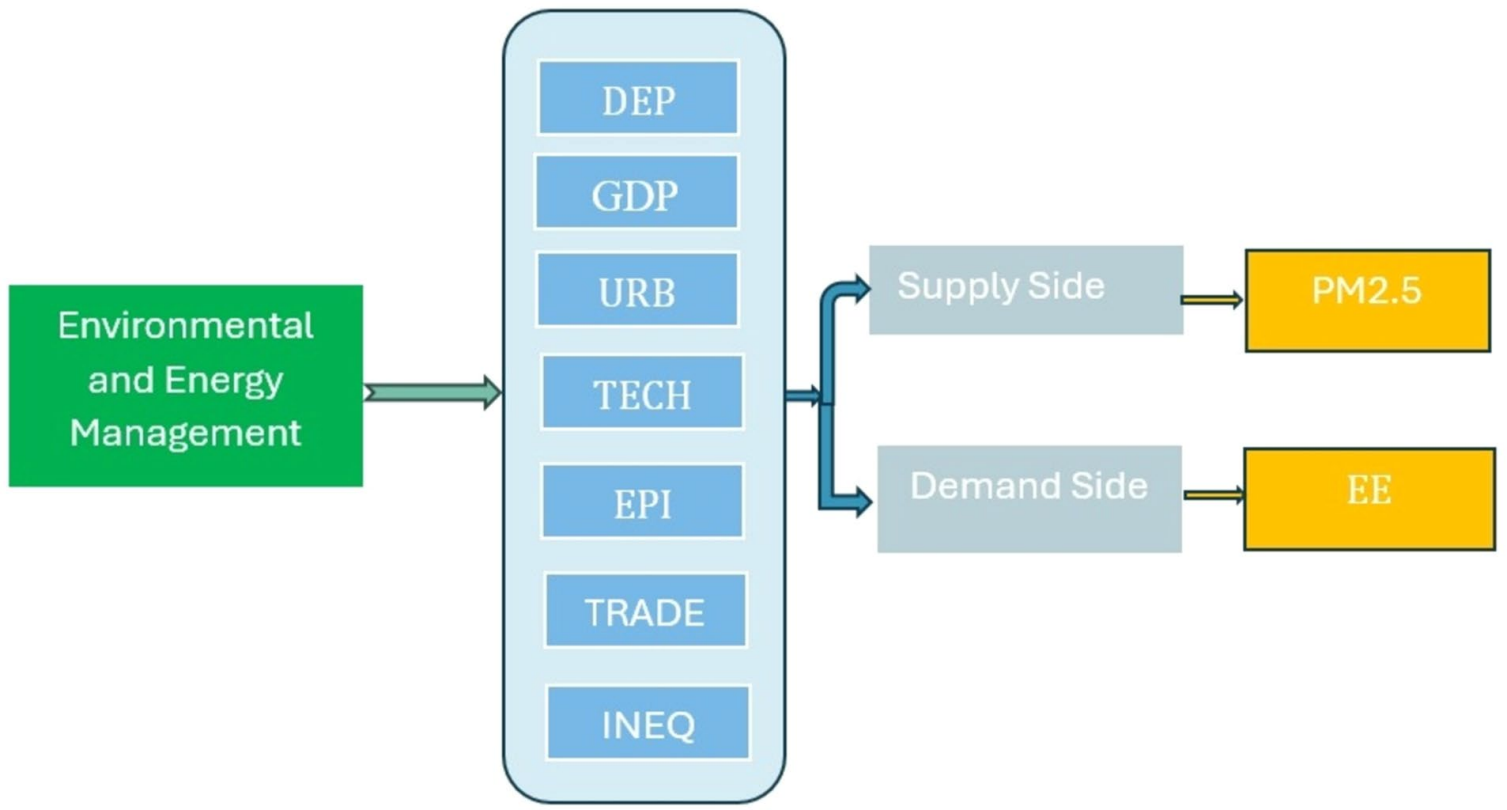
Theoretical framework.

Furthermore, our study investigates the complex correlation among DES, energy efficiency, and environmental quality, with a particular focus on the influence on PM2.5 levels (refer to Figure 1). Although prior research has addressed the overall impact of DES on air quality, it frequently lacks in-depth analysis of the underlying mechanisms and localized environmental consequences. Our work addresses this deficiency by closely examining the complex interrelationships between energy generation and air quality, taking into account the distinct features of the Nordic region.

Finally, our research seeks to reveal the influence of control variables, such as GDP, urbanization, technology, trade volume, income inequality, and the energy price index, on the connection between DES and our dependent variables, energy efficiency, and PM2.5 levels. These control variables are crucial for comprehending the wider context in which DES function and their impact on energy and environmental parameters. Furthermore, previous studies emphasize the importance of policy and governance in influencing the results of decentralized energy systems (34, 35). Supportive policies and incentives are essential for promoting the widespread use of renewable energy technology. The lack of regulatory flexibility and reluctance to change can hinder the process of decentralizing energy production (36, 37). This study specifically examines the dynamics of decentralized energy systems in the Nordic countries, an area renowned for its expertise in sustainable energy solutions.

The theoretical framework offers a full comprehension of the interaction between DEP and a set of control factors, and their impact on energy efficiency and environmental quality. The framework aims to clarify the intricate connections between these factors and provides a basis for empirical research.

Energy efficiency is a crucial factor in this framework, indicating the level of efficacy in using and conserving energy in the Nordic countries. The concept includes enhancements in energy consumption patterns, technical progress, and the production of sustainable energy (38). High energy efficiency refers to the process of maximizing the utilization of energy resources while minimizing wastage. Another dependent variable is environmental quality, specifically assessed through PM2.5 levels, which refers to the concentration of tiny particulate matter. It functions as a gauge of air quality, which has a direct correlation with human health and the general state of the ecosystem. The fundamental independent variable in this approach is the decentralized energy production (DEP) which is anticipated to have a beneficial impact on energy efficiency and environmental quality by decreasing greenhouse gas emissions, promoting the use of renewable energy sources, and improving energy distribution systems.

One possible explanation is that DEP promotes energy efficiency by encouraging the use of renewable energy sources. Renewable energy technologies including wind turbines, solar panels, and hydropower systems are frequently used in decentralized energy production. They provide a more sustainable and efficient way to generate electricity (17, 39). These technologies are frequently distinguished by their superior conversion efficiencies and reduced energy losses in comparison to conventional power generation methods that rely on fossil fuels. Integrating renewable energy sources into decentralized systems can result in decreased energy wastage, heightened energy productivity, and ultimately improved energy efficiency.

Decentralized energy generation reduces the release of harmful pollutants, specifically PM2.5 particles, which positively impacts environmental quality. Adopting cleaner and renewable energy sources like wind and solar power in decentralized systems reduces the release of particulate matter and other harmful pollutants, which contribute to higher levels of PM2.5 in the atmosphere. Moreover, the implementation of distributed energy solutions, derived from decentralized energy generation, can greatly diminish local pollution. The decrease in pollution not only reduces the health hazards linked to air pollution but also enhances the overall environmental condition (40, 41). Introducing emission control methods in decentralized systems can greatly reduce PM2.5 levels, hence enhancing environmental quality. From this theoretical framework, two hypotheses may be formulated for empirical examination:

First, the study hypothesize (H1) that decentralized energy production (DEP) has a significant positive effect on energy efficiency (EE). Second, the study hypothesize (H2) that decentralized energy production (DEP) has a significant negative effect on PM2.5 levels, indicating that DEP is beneficial to environmental quality in Nordic countries.

The collection of control variables comprises GDP, urbanization, technology, trade volume, income inequality, and the energy price index (EPI).

The study also hypothesizes (H3) that there is a positive correlation between GDP and environmental quality (PM2.5) as well as energy efficiency (EE). An elevated GDP level would result in augmented financial resources for investments in environmentally sustainable and energy-efficient technology.

Urbanization serves as a significant measure of urban growth, with the potential to enhance energy efficiency initiatives while also increasing the likelihood of greater energy usage and environmental challenges. Our proposal suggests that the influence of urbanization (URB) on environmental quality (PM2.5) and energy efficiency (EE) is not consistent and may be influenced by the specific context (H4).

Technology innovation is a direct reflection of the significant progress and creation in the field of technology. According to Wang et al. (42), there is a projected positive correlation between enhanced energy efficiency and improvements in technology. The interaction between these components may be influenced by advancements in energy-efficient appliances, renewable energy technology, and industrial activities. The hypothesis (H5) posits that technology enhances energy efficiency (EE) and environmental quality (PM2.5).

Trade volume is a quantitative indicator that measures the magnitude of international commerce. Energy efficiency may be influenced by both exposure to global sustainable energy practices and the commercial exchange of goods and services that need significant amounts of energy (14). Based on our concept, the trading volume (trading) has the capacity to enhance energy consumption while simultaneously promoting the implementation of energy-efficient technologies. This might have contrasting effects on energy efficiency (EE) and environmental quality (PM2.5).

Income inequality pertains to the allocation of income within a country. The increasing disparity in income levels may lead to an uneven allocation of renewable energy resources and energy-efficient technologies. This imbalance has the potential to impact both environmental conditions and overall energy efficiency (35). Our hypothesis posits that the increase in income inequality (INEQ) might negatively affect the quality of the environment (PM2.5) and the efficiency of energy use (EE) as a result of discrepancies in access and benefits (H7).

Fluctuations in energy costs may have a significant impact on the long-term financial viability of decentralized energy systems, as well as their use patterns. The energy price index is a dependable measure of energy expenses, and changes in energy prices may impact both of these parameters. According to, an increase in the energy price index might decrease the probability of buying energy-efficient gear, leading to negative consequences for the environment. This aligns with the findings of the previously stated studies. Based on our hypothesis, which states that this situation is true, the energy price index (EPI) negatively affects both the environmental quality (PM2.5) and the energy usage efficiency (EE) (H8).

## 3 Econometric methodology

### 3.1 The data

We must measure decentralized energy generation (DEP), a fundamental feature of sustainable energy systems, to assess advancements in this area. This paper expresses DEP as a percentage of the total energy production from distributed renewable sources such as wind, solar, and hydropower. The Norwegian Water Resources and Energy Directorate (NVE), the Swedish Energy Agency, and the Danish Energy Agency (43) are among the data sources for distributed energy generation among the Nordic nations.

Air quality is another crucial element that influences human well-being and health. Since PM2.5 is one of the main pollutants tracked and stated as the air quality index (AQI), in this research its concentration serves as an indicator of air quality. PM2.5 refers to fine particulate matter with a diameter of 2.5 micrometers or less, which can penetrate deeply into the respiratory system and potentially lead to negative health consequences. PM2.5 readings between 0 and 50 indicate a green region, meaning good air quality with minimal or no danger. While the range of 101–150 and 151–200 reflect poor and somewhat unhealthy quality correspondingly, the range of 51 to 100 reflects moderate and acceptable quality.

While GDP is computed in inflation-adjusted prices, energy efficiency is a fundamental quality of sustainable energy systems and is expressed as the GDP per unit of energy usage. Urbanization (URB) is yet another important factor affecting the energy consumption patterns of a country. Data is compiled from Usman et al. (15) using a measure based on a nation’s urban population share. Patent awards measuring technology (TECH) allows us to grasp its degree of influence on economic growth and environmental sustainability. The data come from the European Patent Office (EPO). Trade volume (TRADE) is a key economic indicator of Nordic country regional economic activity. The World Trade Organization offers the data source; it is stated as the total value of imports and exports, as a percentage of GDP. Income inequality (INEQ) is a significant socioeconomic indicator indicating the income distribution within a country. World Bank supplies the data for this study, which makes use of the Gini coefficient as indicator. The Energy Price Index (EPI) and the International Monetary Fund (IMF) provide the statistics on relative price changes of energy commodities over time against a base year or period.

### 3.2 The cross-sectional dependence (CD) test

Examining the dynamic interaction across Nordic economies reveals the diverse character of the variables, so selecting the suitable estimator becomes crucial. Examining the possible cross-correlation effects among the chosen indicators—including decentralized energy production (DEPit), GDP (GDPit), urbanization (URBit), technology (TECHit), trade volume (TRADEit), income inequality (INEQit), and the energy price index (EPIit)—due of the interconnections among these economies is absolutely vital. Using a cross-sectional dependence (CD) test, proposed addressing this problem. This test allows us to investigate, across higher and lower middle-income nations, the null hypothesis (H0) of cross-sectional independence against cross-sectional dependence.


(1) $$CD={\left(\frac{TN\left(N-1\right)}{2}\right)}^{\frac{1}{2}}\hat{P}$$

This study depends on the pair-wise correlation and the cross-sectional residual term resulting from the Augmented Dickey-Fuller (ADF) test regression. The Equation (1) combines the cross-sectional dimensions shown as “n” with the dimensionality of time, written as “T.”

### 3.3 Panel unit-root test

Cross-sectional dependence (CD) of variables necessitates careful study of the sequence of integration to avoid erroneous conclusions in regression analysis. Within the framework of a large dataset, the Cross-Sectional Im, Pesaran, Shin (CIPS) second-generation unit-root test is particularly appropriate for identifying cross-sectional dependence or common correlation effects.


(2) $$\Delta Y_{\mathrm{it}}=\alpha_{i}+\beta_{i}Y_{\mathrm{it}-1}+\vartheta_{i}{\bar{Y}}_{t-1}+\delta_{i}\Delta {\bar{Y}}_{t+\varepsilon it}$$

In the above Equation (2), Δ implies the change parameter, *Y* is estimated regressed indicator, ${\bar{Y}}_{t}$ and ${\bar{\Delta Y}}_{t}$ illustrate the $\frac{1}{N}\overset{N}{\underset{i=1}{\sum }}Y_{\mathrm{it}}$ and $\frac{1}{N}\overset{N}{\underset{i=1}{\sum }}\Delta Y_{\mathrm{it}}$ , respectively. εit means the error mechanism.

### 3.4 The system generalized method of moments (SYS-GMM)

In this paper, panel data is estimated using the System Generalized Method of Moments (SYS-GMM). Particularly suitable for tackling unobserved heterogeneity and endogeneity in panel datasets, SYS-GMM is an expansion of the conventional Generalized Method of Moments (GMM) (44). Many elements contribute to the main cause of unobserved variability in the sample: population, technology, and resource levels among others. Either removing the mean value from certain variables or using a first-difference technique will help to address this unobserved variability. Subtracting the mean value, especially in a situation with many cross-sections (N) and limited time series (T) (N > T), might therefore result in the development of regressors that are not independently distributable from the error term (45) put forth a revolutionary solution—the first-difference approach—to address this problem. This method uses one or more lagged dependent variables as regressors to account for the partial adjustment mechanism and estimators especially designed for modeling dynamic panel data. For the energy efficienty and PM2.5 Level, the equation for System-GMM at level and at first difference is thus as follows.

### 3.5 Artificial neural network

ANN is a type of machine learning model inspired by the structure and functioning of the human brain. ANN is commonly used in computer vision for Natural language processing, image recognition, speech recognition, and pattern recognition. Due to the excel in learning complex and nonlinear mapping ANN emerged as widely used machine learning algorithms. In recent years, the contribution of ANN technique in forecasting and predicting macroeconomics data is significantly increased due its accuracy and computational speed (46). ANN is defined by “a massively parallel distributed processor made up of simple processing units, which have a neural propensity for storing experimental knowledge and making it available for use.” ANN are computational models that are based on connected neurons following some architecture. Communication among neurons is performed via signals. The neurons perform calculation based on the provided training.

In ANN the data is passed through the input layer and this data is further processed to the hidden layer and the first hidden layer performs some operation on the input layer and sends the information to the second layer. The second layer in ANN captures non-linearity and expressiveness. In each layer the non-linear activation function is applied to the linear function inputs, which incorporates the non-linearity in the model. The function of non-linear activation is to learn and approximate complex, relation of non-linear mapping between input and output of the model. In ANN the neurons receive the knowledge by learning process by using synaptic weights which are stored in interneuron connection. The hidden neurons quantity has an influence on the prediction accuracy and training speed, the rules-of-thumb suggesting a balance (47). The more hidden layer increases the capacity of neural nets to model intricate relationships in the data by which the networks learn and remove the noisy information and focus on the important features. Finally, the results are passed from the final layer to the output node.

**Figure 2:**
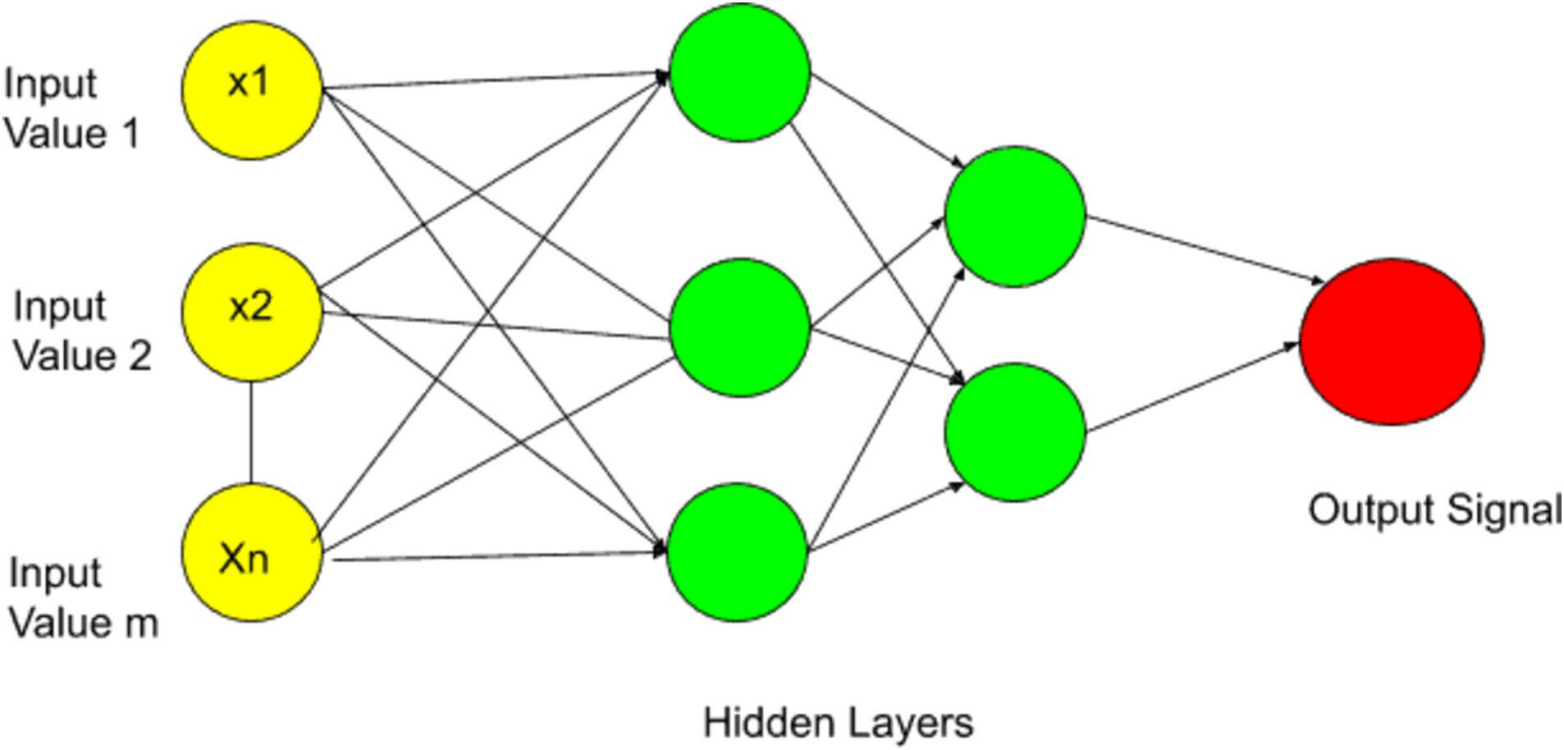
ANN model network graph.

In this model we have used ANN with seven input features and two hidden layers each with seven nodes (see Figure 2). A sequential neural network model is built using Keras with three layers: 64 neurons in the first hidden layer, 32 neurons in the second hidden layer, and 1 neuron in the output layer. Rectified Linear Unit (RELU) is used which captures non-linear features important for modeling intricate relationships. In this paper we use the Adam optimizer (48) which is widely used in ANN, it converges quickly while saving computational cost. The RELU function is used as an activation function. To enhance the training effectiveness normalization was performed. To enhance the effectiveness of training and to provide shorter training time, a standard scalar is applied on the dataset. The dataset is split into two parts training dataset and test datasets. Eighty percent of the data was used for network training and 20 % was used for testing, i.e., to calculate the prediction accuracy of the model. The training and testing accuracy is calculated. The prediction of ANN accuracy is calculated using the Mean square error, Root Mean square error, Absolute Mean square error, and R square.

The mathematical model of ANN consists of feed-forward pass through the network, which involves the neuron activation and the final output calculation. The mathematical model of ANN with two hidden layers is expressed below. Let $\left(x_{1},x_{2},\ldots ,x_{n}\right)$ be the input features.


$$z_{1}^{1}=w_{11}^{1}x_{1}+w_{21}^{1}x_{2}+\ldots +w_{n1}^{1}x_{n}+b_{1}^{1}$$

$$a_{1}^{1}=\mathrm{ReLU}\left(z_{1}^{1}\right)$$

$$z_{1}^{2}=w_{11}^{2}a_{1}^{1}+w_{21}^{2}a_{2}^{1}+\ldots +w_{m1}^{2}a_{m}^{1}+b_{1}^{2}$$

$$a_{1}^{2}=\mathrm{ReLU}\left(z_{1}^{2}\right)$$

$$z_{1}^{\mathrm{output}}=w_{11}^{\mathrm{output}}a_{1}^{3}+w_{21}^{\mathrm{output}}a_{2}^{3}+\ldots +w_{q1}^{\mathrm{output}}a_{q}^{3}+b_{1}^{\mathrm{output}}$$

$$\hat{y}=z_{1}^{\mathrm{output}}$$

Here $\left(w_{ij}^{k}\right)$ represents the weight associated with the connection between the i th neuron in layer (k-1) and the j th neuron in layer (k). the $b_{j}^{k}$ is the bias term for the j th neuron in layer (k) $\left(a_{j}^{k}\right)$ is the output (activation) of the j th neuron in layer (k). $\left(\mathrm{ReLU}\left(\cdot \right)\right)$ is the Rectified Linear Unit activation function.

Furthermore, we have used Adam optimizer which combines ideas from RMsprop and Momentum. The update rule for the parameter θ in the adam optimizer is following.

1. Initialize the optimizer $m_{0}=0$ , $v_{0}=0$ . Here, m is the first moment estimate (mean) and v is the second moment estimate which we can called as uncertain variance.
2. Update the parameters.

$$m_{t}=\beta_{1}m_{t-1}+\left(1-\beta_{1}\right)\nabla J\left(\theta_{t}\right)$$

$$v_{t}=\beta_{2}v_{t-1}+\left(1-\beta_{2}\right)\nabla J{\left(\theta_{t}\right)}^{2}$$

3. In the above equation $\nabla J\left(\theta_{t}\right)$ is the objective function gradient with respect to the parameters $\beta_{1}$ , $\beta_{2}$ , and θ .
3. Correction of Bias:
${\hat{m}}_{t}=\frac{m_{t}}{1-\beta_{1}^{t}}$ and ${\hat{v}}_{t}=\frac{v_{t}}{1-\beta_{2}^{t}}$ this bias-corrected estimate is utilized to keep the moment estimates away from being biased approaching to zero, notably in early time steps.
4. Finally, the parameters update

To find the predictive accuracy of the random model and measure its effectiveness following accuracy test are performed. We calculated the Root Mean Square Error (RMSE), the Mean Absolute Error (MEA), Mean Square Error (MSE), and finally R-Squared (R2, coefficient of determination). Following are the formulas of the test performed.


$$\mathrm{RMSE}=\sqrt{\overset{n}{\underset{i=1}{\sum }}\frac{\hat{y_{i}}-y_{i}}{n}}$$

$$MEA=\frac{1}{n}\overset{n}{\underset{i=1}{\sum }}\left|\hat{y_{i}}-y_{i}\right|$$

$$MSE=\frac{1}{n}\overset{n}{\underset{i=1}{\sum }}{\left(\hat{y_{i}}-y_{i}\right)}^{2}$$

$$R^{2}=1-\frac{\sum_{i=1}^{n}\left(\hat{y_{i}}-y_{i}\right)}{\sum_{i=1}^{n}\left(\hat{y_{i}}-y_{i}\right)}$$

### 3.6 The econometric models


$$\begin{array}{l} \mathrm{EEit}=\beta 1\mathrm{DEPit}+\beta 2\mathrm{GDPit}+\beta 3\mathrm{URBit}+\\ \beta 4\mathrm{TECHit}+\beta 5\mathrm{TRADEit}+\beta 6\mathrm{INEQit}+\\ \beta 7\mathrm{EPIit}+\alpha i+\tau t+\epsilon it---\left(\mathrm{Model}-1\right) \end{array}$$

Here, Energy Efficiency (EEit) is hypothesized to be influenced by decentralized energy production (DEPit), GDP (GDPit), urbanization (URBit), technology (TECHit), trade volume (TRADEit), income inequality (INEQit), and the energy price index (EPIit). While *β*1, β2… β7 are the parameters to be estimated. To capture the dynamics over time, we create first-difference equation:


$$\begin{array}{l} \Delta \mathrm{EEit}=\beta 1\Delta \mathrm{DEPit}+\beta 2\Delta \mathrm{GDPit}+\beta 3\Delta \mathrm{URBit}+\beta 4\Delta \mathrm{TECHit}+\\ \beta 5\Delta \mathrm{TRADEit}+\beta 6\Delta \mathrm{INEQit}+\beta 7\Delta \mathrm{EPIit}+\Delta \epsilon it----\left(\mathrm{Model}-2\right) \end{array}$$

$$\begin{array}{l} \mathrm{PM}2.5\mathrm{it}=\delta 1\mathrm{DEPit}+\delta 2\mathrm{GDPit}+\delta 3\mathrm{URBit}+\delta 4\mathrm{TECHit}+\\ \delta 5\mathrm{TRADEit}+\delta 6\mathrm{INEQit}+\delta 7\mathrm{EPIit}+\alpha i+\tau t+\nu it---\left(\mathrm{Model}-3\right) \end{array}$$

PM2.5 level (PM2.5it) is also hypothesized to be influenced by DEPit, GDPit, URBit, TECHit, TRADEit, INEQit, and EPIit. While *δ*1, δ2… δ7 are the parameters to be estimated. To capture the dynamics over time, we create first difference equation


$$\begin{array}{l} \Delta \mathrm{PM}2.5\mathrm{it}=\delta 1\Delta \mathrm{DEPit}+\delta 2\Delta \mathrm{GDPit}+\delta 3\Delta \mathrm{URBit}+\delta 4\Delta \mathrm{TECHit}+\\ \delta 5\Delta \mathrm{TRADEit}+\delta 6\Delta \mathrm{INEQit}+\delta 7\Delta \mathrm{EPIit}+\Delta \nu it---\left(\mathrm{Model}-4\right) \end{array}$$

We selected the instrument (Zit) for DEPit as the lagged level which is correlated with DEPit but not with the error term εi or νi. The SYS-GMM estimator simultaneously estimates the equations in levels and differences and uses the instruments to account for endogeneity. It also includes country-specific fixed effects (*α* i) and time-specific fixed effects (*τ* t) to control for unobserved heterogeneity. The SYS-GMM estimator will produce coefficient estimates for β and δ, representing the relationships between the variables, while addressing endogeneity and unobserved heterogeneity. The standard errors should be robust and can account for heteroskedasticity and serial correlation in the data.

## 4 Results and discussion

It is crucial to carefully consider the order of integration of the variables included in this study before diving into an analysis of the long-term effects of decentralized energy generation on energy efficiency and environmental quality. It is imperative to take this preventive measure in order to avoid spurious regression from emerging. Many methods for panel unit root testing have been proposed in the literature; these include those by (49–51), among others. Because of their established track record of producing more dependable and consistent findings, we have chosen to use the unit root tests created Im et al. (CIPS) second-generation unit-root test is appropriate for identifying the order of integration in the selected variables.

The Unit Root Test findings are given in Appendix A. Every variable is integrated at order 1(1), according to the results of the second-generation unit-root test, and this includes a constant, intercept, and trend component. As a result, we have decided to use initial differences in a two-step system GMM estimator.

After the affirmation of the existence of CD (see Appendix 1) and the proper order of integration [deviated from *I* (2)] among the variables, utilization of the System GMM approach for estimation is supported by the econometric theory (31). Table 1 presents a thorough summary of the descriptive statistics related to the chosen variables, revealing information about their degrees of variability and primary trends. In the meanwhile, Table 2 shows these variables’ Pearson correlation coefficients. Interestingly, decentralized energy production shows an inverse link with PM2.5 levels but a strong positive correlation with energy efficiency. This finding implies that decentralized energy generation improves environmental quality and has a positive effect on energy efficiency. Moreover, it is important to note that none of the variables we have selected have multicollinearity as we continue with our regression study.

**Table 1:** Descriptive statistics.

Variables	Mean	SD	Min	Max
EEit	300.0	35.98	240.0	470.0
DEPit	0.320	0.412	0.224	0.456
GDPit	442.5	34.76	15.22	688.2
URBit	48.00	3.123	43.00	59.93
TECHit	210.0	80.00	79.00	350.0
TRADEit	40.00	65.00	35.00	75.89
INEQit	29.99	11.34	22.34	35.45
EPIit	106.32	34.99	102.2	110.2

**Table 2:** Pearson correlation coefficients.

Variables	EEit	DEPit	GDPit	URBit	TECHit	TRADEit	INEQit
EEit	1						
DEPit	0.73	1					
GDPit	0.51	0.35	1				
URBit	0.58	0.48	0.44	1			
TECHit	0.45	0.39	0.62	0.05	1		
TRADEit	0.61	0.27	0.41	0.42	0.53	1	
INEQit	−0.39	−0.11	−0.34	0.25	0.10	0.13	1

Our research has produced an interesting result with a positive coefficient for the influence of decentralized energy production (DEP) on energy efficiency and a negative coefficient for PM2.5 level (Table 3). This implies that the amount of money spent on decentralized renewable energy sources, including wind, solar, and hydropower, has a quantifiable correlation with PM2.5 pollution reduction and energy efficiency. This result accords with other studies on the benefits of renewable energy sources for the environment (13). This result is particularly critical given the Nordic countries, whose legislative actions have revolved around sustainability and renewable energy.

**Table 3:** Result of system GMM.

Variables	Energy efficiency	PM2.5 Level
DEPit	1.07**	−0.28**
S.E	0.431	0.101
GDPit	0.89**	0.375*
S.E	0.263	0.166
URBit	0.23*0	0.152*
S.E	0.011	0.070
TECHit	0.358*	−0.261*
S.E	0.135	0.0887
TRADEit	0.43**	0.211*
S.E	0.11	0.099
INEQit	−0.27*	0.002
S.E	0.125	0.87
EPIit	−0.34*	0.220*
S.E	0.016	0.111
Constant	4.11*	5.55*
S.E	0.771	3.011
R2	65	0.64
$J.\mathrm{stat}\;\left(p\right)$	0.40	0.50
No.Instruments	1	1
$AR1\;\left(P\right)$	0.25	0.136
$AR2\;\left(P\right)$	0.59	0.72
$\mathrm{Sargen}\;\mathrm{Test}\left(P\right)$	0.69	0.73
Observations	100	100

*, **, *** shows the significance level at 1, 5, and 10%, respectively. S.E shows standard errors. All explanatory variables are lagged by one year.

The theory that the deployment of dispersed decentralized renewable energy sources might lead to higher energy efficiency matches the shown positive correlation. These sources contribute to reduce PM2.5 pollution by means of decreased environmental effect and emissions. Given that renewable energy technologies provide a sustainable substitute for conventional fossil fuels, which are linked with more emissions and worse energy efficiency, this link appears reasonable. Studies supporting our conclusions include Ur Rehman et al. (17), who claimed that lowering environmental pollutants align with investments in renewable energy.

In our study, country-specific and general fixed effects have statistically significant value. Time-fixed effects underline the importance of time-based elements influencing variables included in our study, which could have changed with time and influenced results. National fixed effects, however, show the unique dynamics and traits of the Nordic countries on the panel. It suggests that country characteristics rather than random oscillations explain differences among nations. Time and country fixed effects highlight how country-specific and chronological factors influence environmental quality and energy economy. Knowing these effects will enable researchers in Nordic region regarding sustainable energy and to identify intricate connections between elements of interest over time within every nation.

According to the results of the study, urbanization and energy efficiency show a significant and positive link. Furthermore technological advancement has significant and positive impact on energy efficiency a negative impact on PM2.5 level. More precisely, the findings imply that advances in energy efficiency and declines in PM2.5 concentrations are directly linked to raising the capital expenditure on research and development in the area of technology. For a good length of time, the dual effects of urbanization on environmental quality and energy efficiency have been acknowledged in the corpus of scholarly work. Urbanization might be a focus point for environmental issues and help to promote energy efficiency at the same time (52). Kennedy et al. (53) highlight the complex interaction between urbanization and energy efficiency, therefore highlighting the importance of thorough urban planning and sustainability policies.

The positive correlation between energy efficiency and technology is primarily the development of more energy-efficient systems through technological innovation. The positive correlation between technological investment and energy efficiency emphasizes the need to invest in R&D and technological innovation (46). The potential increase in PM2.5 pollution due to urbanization emphasizes the importance of proper urban planning to create a friendly environment. Our analysis reveals a significant relationship between PM 2.5 levels and energy efficiency and trade volumes. There are two possible explanations for this. First, it emphasizes how trade between countries increases energy efficiency and how globalization helps to develop sustainable energy solutions. Second, it acknowledges that the increased commercial volume could potentially be due to emissions from transportation, leading to increased levels of PM 2.5 pollution (17).

The result of the study also revealed that increase in high energy prices are negatively associated with energy efficiency and add to environmental deterioration by increasing the level of PM2.5 pollutant. Islam and Miao (17) argued that the higher the level of energy price the lover will be the level of investment in energy efficient practices. Such increase on from one side decrease energy efficiency and on the other hand increase the level of pollution.

We conducted a thorough analysis of different model specifications using well-established econometric approaches, such as the Arellano-Bond and Blundell-Bond Generalized Method of Moments (GMM), in order to verify the validity of our study findings. The coefficient and standard error did not change significantly giving us confidence in the validity and dependability of our findings.

### 4.1 Artificial neural network results

**Figure 3:**
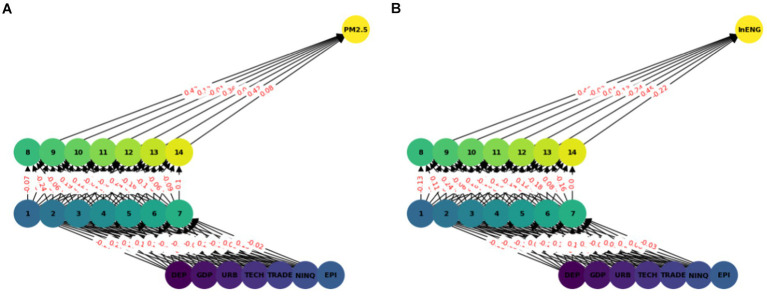
ANN analysis Denmark. **(A)** PM2.5 ANN analysis, **(B)** Energy efficiency.

**Figure 4:**
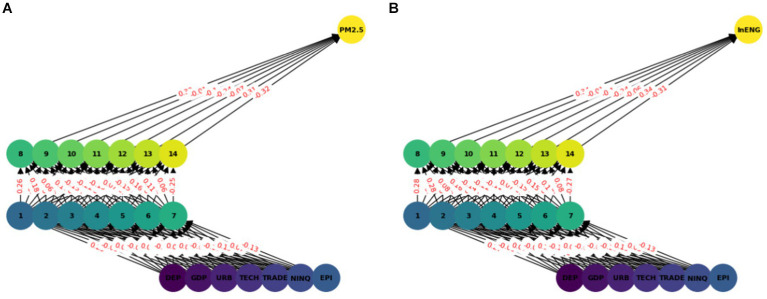
ANN analysis Sweeden. **(A)** PM2.5 ANN analysis, **(B)** Energy efficiency.

**Figure 5:**
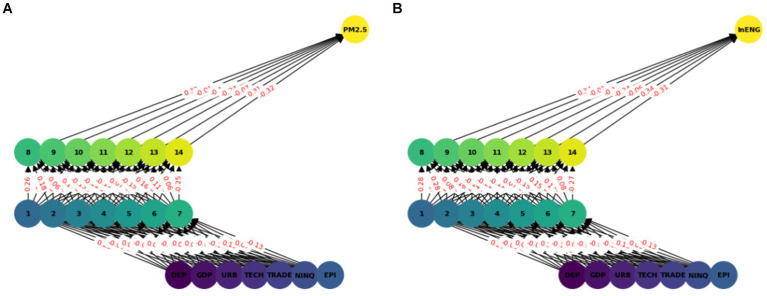
ANN analysis Finland. **(A)** PM2.5 ANN analysis, **(B)** Energy efficiency.

**Figure 6:**
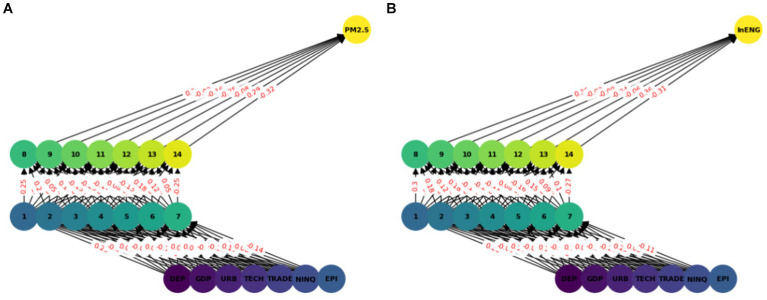
ANN analysis Norway. **(A)** PM2.5 ANN analysis, **(B)** Energy efficiency.

**Figure 7:**
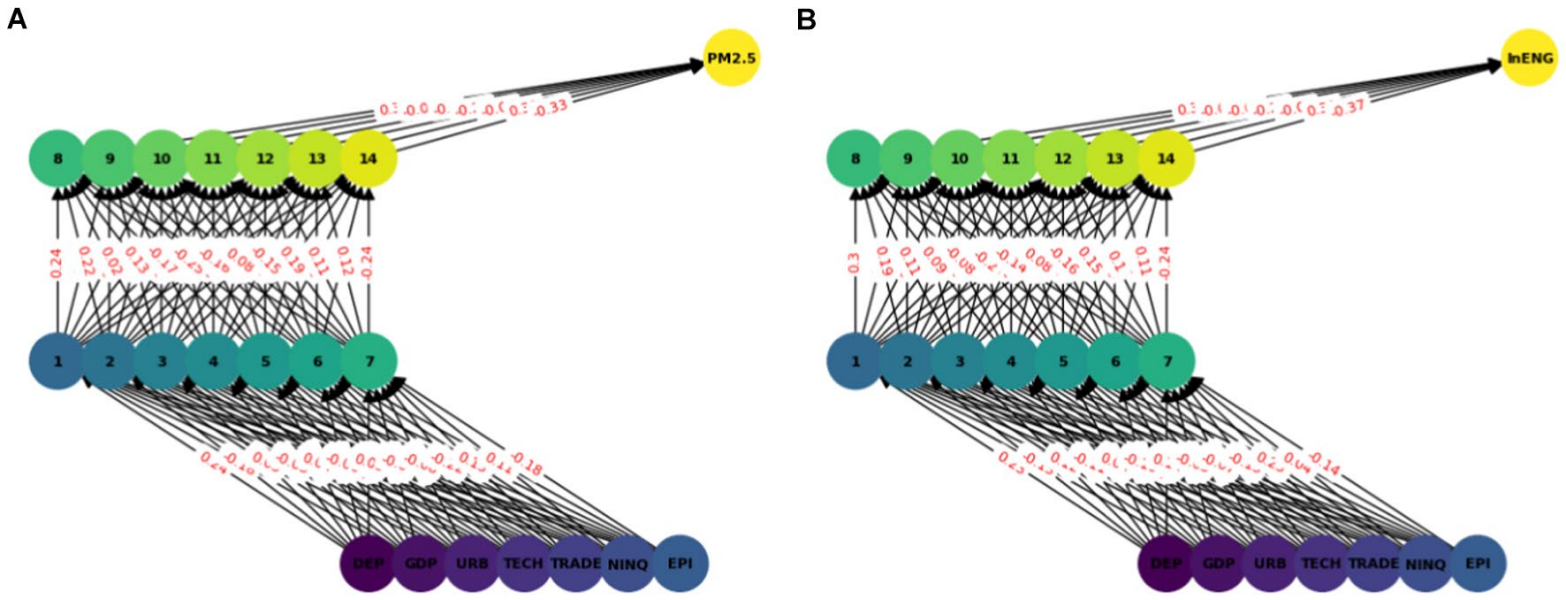
Combined ANN analysis. **(A)** PM2.5 ANN analysis, **(B)** Energy efficiency.

The multilayer ANN model was developed to test the PM2.5 and ENG predictability using the seven parameters. The data was split into test and train as described earlier. We tested each country separately with the following seven inputs and PM2.5 and EE as output. We predicted the PM2.5 for Denmark, Sweeden, Norway, and Finland and combining all the countries. In this model, two hidden layers are constructed, each with seven neurons. The reason for selecting the seven neurons is the number of input variables. The Neuron networks are plotted from Figures 3–7 in (a) PM2.5 is plotted for each country and in (b) EE is plotted for each country. Furthermore, we have applied some statistical measures to predict the accuracy of the model, in Table 4, values associated with PM2.5 are shown and in Table 5, ENG are described. From these Tables it is very clear that we have achieved high accuracy. As discussed above we have applied the following four methods Root Mean Square Error (RMSE), the Mean Absolute Error (MEA), Mean Square Error (MSE), and finally R-Squared (R2, coefficient of determination).

**Table 4:** Results of PM2.5 using ANN.

Countries	RMSE	MEA	MSE	*R*-Squared
Sweeden	0.077	0.065	0.006	0.570
Denmark	0.061	0.053	0.004	0.270
Norway	0.044	0.035	0.002	0.046
Finland	0.324	0.301	0.105	0.233
Combined	0.121	0.070	0.015	0.932

**Table 5:** Results of energy efficiency using ANN.

Countries	RMSE	MEA	MSE	*R*-Squared
Sweeden	0.449	0.358	0.202	0.570
Denmark	0.386	0.297	0.149	0.196
Norway	0.485	0.406	0.236	0.448
Finland	0.833	0.743	0.694	0.840
Combined	0.335	0.269	0.112	0.902

## 5 Conclusion

To understand the complex relationships between decentralized energy components and how they affect energy efficiency and environmental quality, we conducted an imperial study in the Nordic Region. We found important information by examining and interpreting data carefully, in terms of urbanization, technology, GDP, trade volume and energy efficiency. These findings highlight the complex relationship between decentralized energy production, energy efficiency and environmental quality. As PM 2.5 says. Our study, based on a distinct sample of Nordic countries including Norway, Sweden, Denmark and Finland, allowed us to find appropriate geographical results. Since our panel data is dynamic and endogeneity issues are considered, the GMM and ANN systems have helped ensure our study power.

Our analysis found many connections that show the complexity of factors. A positive correlation between urbanization (URB) and energy efficiency suggests that a higher rate of urbanization improves energy efficiency, but urban populations may increase PM 2.5 levels due to pollution. Additionally, Energy efficiency is positively associated with increase in R&D costs as a percentage of GDP because technology (TECH) is a key factor in energy efficiency and environmental improvement. Energy efficiency has increased by GDP and shows that economic growth contributes to energy-efficient methods and technologies. Trade volume (TV) has had a positive impact on energy efficiency and highlighted the role of global trade in the expansion of energy-efficient technologies and practices. On the other hand, large volumes of trade can lead to increased energy consumption and pollution, so environmental trade is very important. This research reveals many factors that affect Norway’s environmental quality and energy efficiency. Negative associations propose plans and investments in energy and pollution reduction. Complex results show the need for comprehensive solutions and mechanisms to solve the balance in environmental quality due to trade, economic expansion and urbanization. Finally, our studies emphasize the need for a comprehensive and situational energy and environmental control project program. We need to consider balances and combinations to develop methods that increase environmental quality and energy efficiency.

### 5.1 Policy implications

The policy implications of our work emphasize the need for conversion to renewable energy sources including air, sun, and water. Policymakers should provide tax structure regarding the purchase of energy efficient production process and support sustainable energy technology to minimize pollution and boost energy efficiency. Urbanization (URB) promotes sustainable urban development, so policies should support urban transit and infrastructure to decrease environmental issues. Encouragements are needed to boost technology and R&D in numerous fields to eliminate imbalances and promote environmentally friendly economic growth. Additionally, policies should be formulized for sustainable international trade and recognize transport pollution’s environmental effect.

Additionally, Norwegian officials should address regional cooperation and the necessity for a comprehensive and unique local plan to enhance environmental quality and energy efficiency in the Northern Region given the link between energy and environmental challenges. Sustainable practices, technology and innovation, and regional collaboration may help these nations develop a greener, more energy-efficient future.

## Data Availability

The raw data supporting the conclusions of this article will be made available by the authors, without undue reservation.

## Appendix 1

**Table A1:** Cross-sectional dependence and order of integration.

Variables	CD-test	CIPS	
		Level	First difference
EEit	38.468***	−3.963**	−11.844***
DEPit	17.620***	−2.677**	−7.601***
GDPit	19.502**	−1.035	8.330***
URBit	20.600***	−2.865***	−10.199***
TECHit	14.131***	−1.088	−11.084***
TRADEit	9.361***	−1.841	−9.801***
EEit	25.27***	−1.354**	−10.129***

*, **, *** display significance level at 10, 5, and 1%, respectively.
